# Editorial: Novel insights in insect immunity: regulators, signaling, and mechanisms

**DOI:** 10.3389/fimmu.2026.1999950

**Published:** 2026-10-05

**Authors:** Xin Hu, Aneta Strachecka, Fei Wang, Hong Zhou, Kui Zhang, Ioannis Eleftherianos, Amr Mohamed

**Affiliations:** 1 Department of Cancer Biology, The University of Texas MD Anderson Cancer Center, Houston, TX, United States; 2 Department of Invertebrate Ecophysiology and Experimental Biology, Faculty of Environmental Biology, University of Life Sciences in Lublin, Lublin, Poland; 3 State Key Laboratory of Resource Insects, Medical Research Institute, Southwest University, Chongqing, China; 4 USDA/ARS Chlidren’s Nutrition Research Center, Department of Pediatrics, Baylor College of Medicine, Houston, TX, United States; 5 The Pritzker School of Molecular Engineering, The University of Chicago, Chicago, IL, United States; 6 School of Biological Sciences, Queen’s University Belfast, Belfast, United Kingdom; 7 Department of Entomology, Faculty of Science, Cairo University, Giza, Egypt

**Keywords:** immune priming, immunometabolism, innate immune responses, insect immunity, insect physiology, insect-pathogen interactions, neuroendocrine regulation, pest management

Insects occupy diverse ecological niches and include species of major importance to agriculture and public health. Their survival depends on innate immune mechanisms that recognize biological threats, coordinate protective responses and maintain physiological integrity. These mechanisms connect pathogen recognition with intracellular signalling and cellular defences, including antimicrobial peptide production, phagocytosis, encapsulation and melanization. The Toll, immune deficiency (IMD), JAK/STAT, JNK and STING signalling pathways contribute to this regulatory network, with their respective roles varying among insect species, tissues and infection contexts (**Figure 1**). Effective immunity requires coordination between recognition, signalling and effector responses to achieve pathogen control while preserving tissue homeostasis and survival (1). Understanding how these responses are regulated is therefore essential for explaining host-pathogen interactions and identifying opportunities to manage pests and disease vectors.

**Figure 1:**
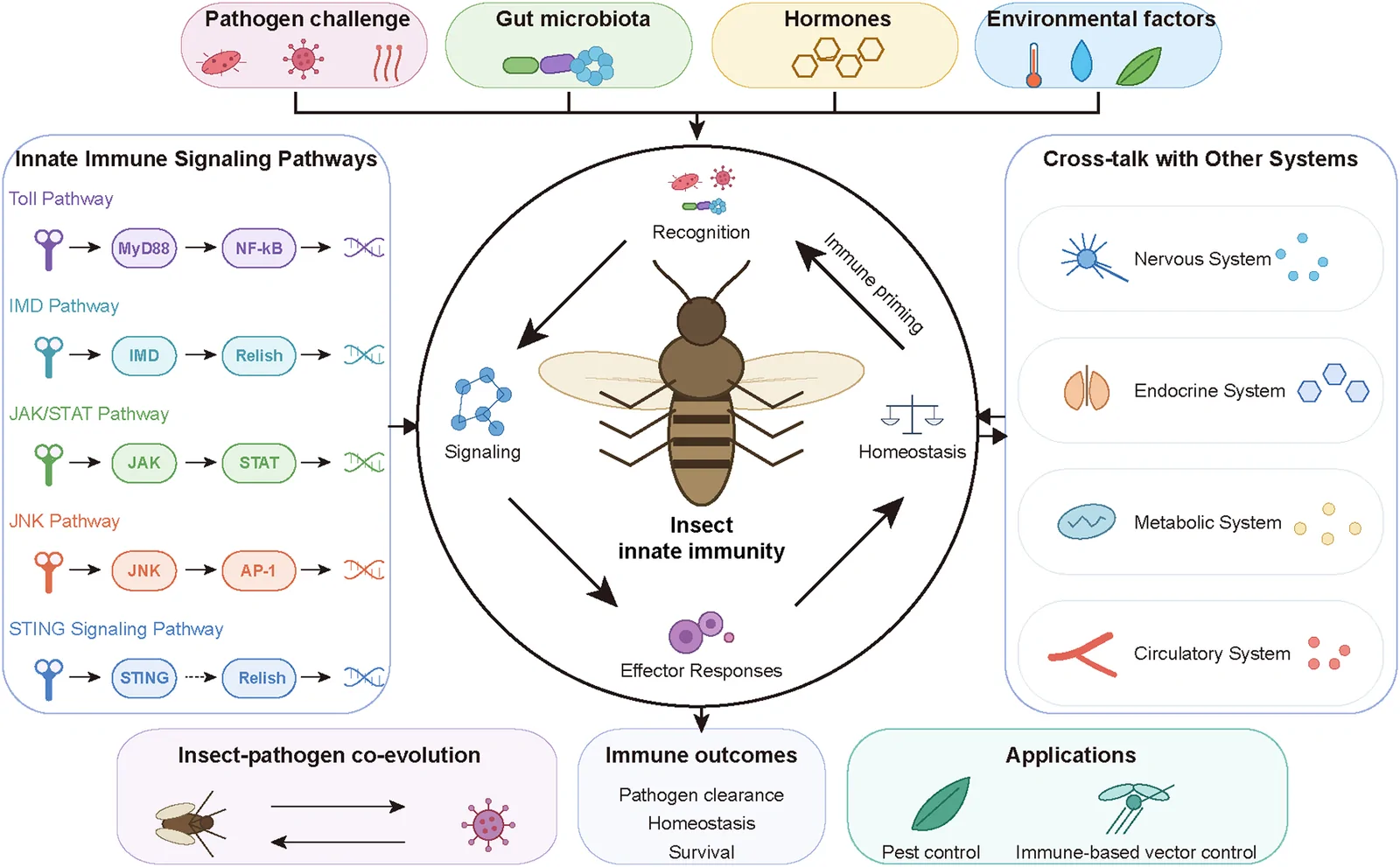
Regulatory networks and physiological crosstalk in insect innate immunity.

Immune responses are also shaped by the broader physiological and environmental contexts in which infection occurs. Gut microbiota, hormonal signals and environmental conditions influence immune activity, while interactions with the nervous, endocrine, metabolic and circulatory systems connect defence with organismal function (2). Evidence of immune priming further shows that previous exposure can modify subsequent protection in some insect-pathogen combinations (3). Together, these observations raise questions about how insects discriminate among biological challenges, how immune regulators connect defence with other physiological functions, and how protective responses are adjusted to changing internal and external conditions. Resolving these questions requires linking molecular mechanisms to cellular responses and organismal outcomes.

The articles in this Research Topic approach these questions from complementary perspectives. They examine how responses vary with the challenge, how molecules involved in defence contribute to other host functions, and how communication and resource allocation across physiological systems shape immune responses.

## Challenge-dependent immune responses

Differences between immune responses begin with the challenges insects encounter. In *Mythimna separata*, Ono et al. compared live, axenically prepared *Caenorhabditis elegans* with inert beads and buffer controls. Nematodes elicited slower encapsulation than beads, yet their transcriptional trajectories were qualitatively different, including more sustained Toll-associated gene expression. The distinction therefore extended beyond the speed of the response. Time adds another dimension: Chen et al. identified changing immune-related transcriptional profiles in *Brontispa longissima* following parasitism by *Asecodes hispinarum*, including later reductions in phenoloxidase transcripts. These studies highlight the importance of considering both the challenge and the stage of interaction.

Even within one bacterial species, virulence determinants can alter the host response. Mukherjee et al. found that pathogenicity-island deletions in uropathogenic *Escherichia coli* changed virulence in *Galleria mellonella* alongside antimicrobial-gene expression and global histone-acetylation profiles. A haemolysin-deficient mutant elicited lower histone H4 lysine 5 acetylation than the parental strain in both larvae and human bladder epithelial cells. Together with the preceding studies, this work extends the question of response differences from challenge identity and timing to the pathogen’s genetic repertoire.

## Multifunctional immune regulators

Immune molecules also act beyond defence, shaping other aspects of host physiology. Li et al. identified immulectin 4 (CpIML4) through transcriptomic and proteomic investigation of *Beauveria bassiana* infection in *Conogethes punctiferalis*. RNA interference (RNAi) against CpIML4 accelerated mortality after fungal challenge, while effects on survival and development without challenge suggested a broader physiological role. This functional breadth is also evident in the work of Liu et al.: recombinant isoforms of the secreted C-type lectin PaSCLec from *Periplaneta americana* bound bacterial components, agglutinated bacteria, and enhanced haemocyte phagocytosis *in vitro*; PaSCL-Ad also inhibited bacterial growth. PaSCLec knockdown altered immune transcripts and impaired leg regeneration. The two studies thus connect lectins with microbial defence and with processes that maintain or restore the host.

## Immune regulation in a whole-organism context

Physiological communication across systems is similarly relevant to immune regulation. In *Tenebrio molitor*, Konopińska et al. found that immune stimulation altered tachykinin-related peptide (TRP) and receptor expression, while pharmacological and RNAi interventions changed immune transcripts and phenoloxidase activity in a tissue- and treatment-dependent manner. Neuropeptide signalling therefore provides one point of contact between immune and neural physiology. Endocrine signals offer another: Vieira et al. showed that ecdysone receptor knockdown in *Phlebotomus perniciosus* altered gut immune transcripts, bacterial 16S ribosomal RNA signals, and the temporal pattern of *Leishmania infantum* abundance estimated by reverse-transcription quantitative PCR. Parasite developmental-form distributions also shifted. Together, these findings link neuroendocrine regulation to the physiological environments in which immune responses and infections develop.

Coordination also involves the resources available for defence. Adamo synthesises evidence that insect muscle responds to immune signals, produces immune molecules, and redistributes metabolic resources during immune activation. The resulting costs to muscle performance and behaviour link defence to the wider demands of host fitness. Resource availability can also be influenced from outside the insect. Menezes et al. found that feeding on mice with a diet-induced obese and diabetic phenotype reduced *Aedes aegypti* survival and altered lipid and gut transcriptional profiles. In separate infection assays using blood supplemented with equivalent Zika virus titres, samples from these mice yielded lower midgut viral RNA levels without a significant change in infection prevalence; onward transmission was not measured. These complementary perspectives connect insect defence with both internal resource allocation and the nutritional inputs supplied by another host.

## Emerging questions and future directions

Together, these studies point to several questions that remain open. How environmental and nutritional variation reshapes immune regulation across tissues and life stages is not yet clear, nor is the extent to which immune priming and the physiological trade-offs of defence jointly shape host fitness and pathogen transmission. Tracking molecular, cellular and organismal measures across the course of infection may help distinguish regulatory mechanisms that enhance protection from those that impose physiological costs, and could clarify how immune plasticity contributes to insect-pathogen co-evolution and informs more targeted pest and vector management.

Across the collection, defence emerges as a context-dependent property of the host, shaped by the challenge encountered, the functions of immune regulators and communication among physiological systems. This perspective also opens questions about environmental modulation, immune priming and insect-pathogen co-evolution (**Figure 1**). Linking molecular changes to pathogen burden, host fitness and, where relevant, transmission will clarify which mechanisms support protection and which impose physiological costs. These distinctions are essential for translating insect immunology into targeted pest and vector management.
